# Evaluation of immunization coverage and its associated factors among children 12–23 months of age in Techiman Municipality, Ghana, 2016

**DOI:** 10.1186/s13690-017-0196-6

**Published:** 2017-06-26

**Authors:** Martin Nyaaba Adokiya, Benjamin Baguune, Joyce Aputere Ndago

**Affiliations:** 1 Department of Community Health, School of Allied Health Sciences, University for Development Studies, Box 88, Tamale, Ghana; 2 School of Hygiene, Environmental Health Programme, Ministry of Health, Tamale, Ghana; 3 Department of Nursing, School of Allied Health Sciences, University for Development Studies, Tamale, Ghana

**Keywords:** Immunization coverage, Fully immunized, Predictors, Techiman Municipality, Child, Ghana

## Abstract

**Background:**

In Ghana, Expanded Programme on Immunization administrative coverages are usually high while childhood immunization status remains low. Majority of children do not receive all the recommended 7 vaccines in 15 doses before 1 year of age. Surveys to validate administrative coverages and identify predictors of immunization status are not given the desired attention. Thus, the objective of this study was to evaluate the immunization coverage and its associated factors among children aged 12–23 months in Techiman Municipality, Ghana.

**Methods:**

A cross-sectional cluster survey was conducted among 600 children. Data was collected using semi-structured questionnaire through face-to-face interviews. The tools were pre-tested in three communities with similar characteristics. The mothers/caregivers were interviewed and additional information extracted from child immunization cards. We observed the presence of Bacillus Calmette-Guerin scar on each child. Data was entered, cleaned and analyzed using Statistical Package for Social Sciences (SPSS) version 17.0. Descriptive statistics such as percentages, frequencies and cross tabulations performed using SPSS while bivariate and multivariate logistic regression analysis conducted using Stata 12.1 version to estimate the Odds Ratio of not being fully immunized.

**Results:**

In total, 89.5% (537/600) of the children were fully immunized, 9.5% partially immunized and 1.0% received no vaccine. In the multivariate analysis, the following determinants were significantly associated with the likelihood of being not fully vaccinated (Odds Ratio (AOR) larger than 1) : age of the mother/caregiver 40–49 years (AOR = 0.15, 95%CI = 0.05–0.87) compared to less than 20 years; marital status (compared to never married/single: being married AOR = 0.29, 95%CI = 0.13–0.68), ethnicity (compared to the main ethnic group Akan: Frafra (AOR = 4.71, 95%CI = 146–15.18) and Kusaasi (AOR = 0.09, 95%CI = 0.02–0.51), religion (compared to Islam: Christianity AOR = 0.17, 95%CI = 0.06–0.50), sex of child (compared to male: female AOR = 0.39, 95%CI = 0.19–0.80) and possession of immunization card (compared to those having the card: those without the card AOR = 84.43, 95%CI = 17.04–418.33). Mothers/caregivers aged 40–49 years, being married, Kusaasi ethnic groups, Christian and female child have a higher likelihood of being fully immunized, while Frafra ethnic group and no immunization card have a higher likelihood of not being fully immunized. We found no association between immunization status and child’s relationship to respondent; parity; education; occupation and child’s age.

**Conclusion:**

Immunization status (89.5%) and coverages ranged 92 to 99% of the vaccine doses is high compared to national and regional. Problems of not fully immunized persists and needs urgent attention. Education on immunization should be intensified by health providers. Moreover, disadvantaged populations should be reached with immunization services using out-reach activities.

## Background

In Ghana, routine administrative immunization coverages are usually high while childhood immunization status remains low. Majority of children do not receive all the recommended 15 vaccine doses before 1 year of age. At birth, children are protected against certain diseases due to received maternal antibodies. After birth, breastfed babies continue to receive additional antibodies especially when the colostrum is also given. This protection is only temporary. Thus, children need extended protection through artificial immunization [1]. Immunization is a cost-effective means of preventing communicable diseases. Vaccines are available for many dangerous communicable diseases. This resulted in a significant reductions in morbidity and mortality due to communicable diseases especially among infants and children under 5 years of age. In sub-Saharan Africa (SSA), access to vaccines remains a challenges. In recent years, various policies, donors and partners have rolled-out strategies to improve the accessibility problem. Despite the effort and various investments, infant and child mortality remain unacceptably high in the world especially in developing countries [2].

In 1974, World Health Organization (WHO) launched the Expanded Programme on Immunization (EPI) to make vaccines available to all children worldwide [3]. Ghana launched its EPI in 1978 and became operational nationwide in 1985 with the goal of increasing immunization coverage from 6 to 80% among children <1 year of age against six target diseases: Diphtheria, Tetanus, Pertussis, Poliomyelitis, Tuberculosis, and Measles [4]. The following diseases have been added to the first six: Yellow fever, Haemophilus influenza type B, Hepatitis B, Rotavirus diarrhoea and Pneumonia [5]. Since EPI implementation in Ghana, children <5 years of age mortality has declined progressively but still inadequate. According to 2014 Ghana Demographic and Health Survey (GHDS) report, infant mortality rates have reduced slightly from 64, 50 and 41 deaths per 1000 live births while under five mortality rates also declined from 111, 80 and 60 per 1000 live births over a couple of years 2003, 2008 and 2014 respectively [6].

Generally, improvements have been recorded in health, economic, social and political indicators in Ghana. However, nationwide childhood immunization coverage remains at 77% which is less than the 80% target in 1985. However, the Brong Ahafo Region of Ghana where the study is conducted has 82.2% immunization coverage which is slightly high compared to the national coverage [4, 6]. In order to achieve the EPI target nationwide, knowledge on the factors contributing to the observed coverage disparities in the country are critical. Routine administrative data from the health system is often inaccurate and unreliable including immunization coverages. Quality information on non-vaccination is useful for the design and implementation of interventions for the control and elimination of vaccine preventable diseases [7]. Community-based information on vaccination coverages are essential to guide middle level health managers to prioritized health system activities and implement plans with limited resources [7]. Thus, the objective of this study was to evaluate the immunization coverage and its associated factors among children aged 12–23 months in Techiman Municipality, Ghana.

## Methods

### Study design and setting

A cross sectional cluster survey was conducted in Techiman Municipality of Brong-Ahafo Region, Ghana. Techiman Municipality is one of the twenty seven (27) Municipalities/Districts in the region. It has a land surface area of 649.0714sq.km and a population of 166,497 projected from the 2010 population and housing census. The Municipality constitutes about 6.4% of the region’s total population. It has the highest population density of 256.5 people per square kilometer in the region. Health services are provided by a blend of health facilities (public and private sectors). The facilities include - Community-based Health Planning and Services (CHPS) compounds, maternity homes, clinics, health centers and hospitals. As part of the decentralization concept of the health system, the Municipality is demarcated into seven sub-municipalities to facilitate health services delivery.

### Sampling procedure

A ‘30 × 20’ cluster sampling was used as the sampling method instead of the ‘30 × 7’ recommended by WHO which increased sample size. Cluster sampling method is recommended by WHO as a rapid, simplified and economic sampling method in evaluation of vaccination coverage [8]. The study population covered children aged 12–23 months. According to WHO-EPI recommendation, children aged 12–23 months should be used for evaluating immunization coverage if the immunization schedule ends at 9 months as in the case of Ghana. Ideally, no child should receive immunization after first year of life in the country. In sampling, 20 households from each of the 30 clusters were selected. The first selected household was taken as the starting point for each cluster and then continued to the next nearest household until 20 eligible children were obtained. Mothers/caregivers with children 12–23 months of age were interviewed at their homes using face-to-face interviews. The main data was collected between January and February, 2016.

### Sample size determination

The sample size was calculated using: N = [De × Z^2^ × p (1-p)]/d^2^ [9]. Where, **N** is the sample size, **De** (2) is the design effect, the ratio between the variance from the cluster design to the variance that would be obtained from a simple random sampling [9], **Z** (1.96) is the certainty wanted expressed in the percentage point of normal distribution corresponding to the 2-sided level of significant, **P** (77%) is the immunization coverage of Ghana [6] and d (5%) is the desired width of the confidence interval. Therefore; **N** = [2 × (1.96)^2^ × 0.77× 0.23]/(0.05)^2^ = 545. A non-response rate of 10% was added for a total sample size of 600. Mother/caregiver having at least one child aged 12–23 months was the inclusion criteria. In total, 600 respondents (mothers and caregivers) were interviewed.

### Data collection instrument and procedures

Mothers/caregivers were interviewed using a semi-structured questionnaire. The questionnaire covered respondent characteristic such as education level, occupation, parity, age, and religion as well as child’s age and sex. In addition, childhood immunization information was extracted from child’s card. Each mother/caregiver had to give consent before they were interviewed. Specifically, immunization coverage information was obtained by either child immunization cards and mothers recall (verbal reports). Mothers/caregivers were asked to show the child health record card which contains individual immunization data. If the card was available, the interviewer extracted the information on immunization such as the dose and dates received. When no specific information on the card, mother/caregiver was then asked to confirm or otherwise whether the child had received other vaccinations that were not recorded on the card. If there was no immunization card, or if the mother/caregiver was unable to show the card, child’s immunization information was based on their recall. In case of immunization failure, they were asked to give reasons for the failure. The use of mothers recall was a limitation of the study. Mothers/caregivers recall was prone to systematic error (recall bias) caused by differences in accuracy of the remembering child’s immunization information. The data was collected between January and February, 2016.

### Operational definitions

#### Fully immunized

A child who received 1 dose of Bacillus Calmette-Guerin (BCG), 4 doses of Oral Polio Vaccine (OPV), 3 doses of Pentavalent, 3 doses of Pneumococcal Vaccine (PCV), 2 doses of Rotarix (Rota) and 1 dose each of Measles and Yellow fever vaccines. A child is expected to receive a total of 7 vaccines and 15 doses.

#### Partially immunized

A child who did not receive one or more of the prescribed vaccines doses considered to protect the child from vaccine preventable diseases.

#### Not immunized

A child who has received none of the prescribed vaccines doses considered to protect the child from vaccine preventable diseases.

#### Not fully immunized

A combination of both partially immunized and not immunized.

#### Pentavalent

This is a combination of Diphtheria, Pertussis, Tetanus, Haemophilus Influenza type B and hepatitis B antigens.

#### Age limit for immunization

At age 9 months, each child is expected to have received a total of 7 vaccines (15 doses) to complete his/her immunizations.

### Data processing and analysis

Data entered and cleaned using Statistical Package for Social Sciences (SPSS) version 17.0. Descriptive statistics such as frequencies, percentages and cross tabulations were performed. The dependent variable (immunization status) was recorded as fully immunized (children with completed immunization – 7 vaccines or 15 doses) and not fully immunized (partially immunized or not immunized - < 7 vaccines or 15 doses). Data was imported into Stata 12.1 for bivariate and multivariate logistic regression analysis to determine determinants of not-being fully immunized. The analysis focused on socio-demographic characteristics of mothers/caregiver’s, child’s sex, child’s age, possession of immunization card and child’s relationship of respondent. The multivariate analysis was performed to test associations between the dependent variable (immunization status) and the independent variables as predictors (see details on Table 4). We used SPSS and Stata for the data entry, cleaning and multivariate analysis due to familiarity.

### Ethics approval and informed consent

An introductory letter and approval was received from the School of Allied Health Sciences, University for Development Studies, Tamale, Ghana. In addition, permission letter was obtained upon a written request and explanation of the protocol, methods and questionnaires from the Techiman Municipal Health Directorate. At the individual level, the protocol, methods and approach was explained in English or Twi (main local language) and a written consent was obtained from each respondents of 18 years of age and above before the interview was conducted. Among the few teenagers, consent was obtained through their husbands (those married) or parents (those unmarried). Respondents were informed that participating was voluntary and it was their right to stop at any time. They were also informed of data confidentiality by not using any personal identifiers.

## Results

A total of 600 pairs of mothers/caregivers and children were interviewed. The ages of the children ranged 12 to 23 months. The mean and median ages were 17.2 and 17.0 months respectively. In all, 50.5% of the children were boys. Minority (7.5%) of the mothers had tertiary education as against 18.5% had no formal education. Over half of the respondents were married (52.0%) and 24.0% never married. In terms of religion, 67.5% of the mothers were Christians and 25.5% were Muslims. About 90% of the respondents were the biological mothers. Nearly all children (97.0%) possessed health record cards (Table 1).

**Table 1:** Socio-demographic characteristics of mothers/caregivers, Immunization Coverage Survey, Techiman Municipality, Ghana, 2016

Variable		Frequency	%
Education	No formal education	111	18.5
	Primary school	126	21.0
	JSS/Middle school	252	42.0
	SHS	66	11.0
	Tertiary	45	7.5
	Total	600	100.0
Age	<19 years	57	9.5
	20–29 years	225	37.5
	30–39 years	219	36.5
	40–49 years	66	11.0
	≥50	33	5.5
	Total	600	100.0
Marital status	Never married	144	24.0
	Married	312	52.0
	Divorce	27	4.5
	Separated	114	19.0
	Widowed	3	0.5
	Total	600	100.0
Ethnicity	Akan	282	47.0
	Dagaati	102	17.0
	Frafra	57	9.5
	Kusaasi	66	11.0
	Ewe	39	6.5
	Dagomba	39	6.5
	Others^a^	15	2.5
	Total	600	100.0
Religion	Islam	153	25.5
	Christianity	405	67.5
	Traditionalist	6	1.0
	No Religion	36	6.0
	Total	600	100.0
Occupation	Salary worker	63	10.5
	Trader	258	43.0
	Farmer	132	22.0
	Artisan	99	16.5
	Housewife	48	8.0
	Total	600	100.0
Sex of child	Male	303	50.5
	Female	297	49.5
	Total	600	100.0
Card possession	Yes	582	97.0
	No	18	3.0
	Total	600	100.0
Relationship with child	Mother	540	90.0
	Caregiver	60	10.0
	Total	600	100.0

Others^a^ (Gurisi - 4, Krobo – 3, Wangara – 3, Sisaala – 3, Gruma – 1 and Fulani – 1)

### Immunization coverage by immunization card and mother recall

In total, 89.5% of children were fully immunized (7 vaccines or 15 doses) by time of survey using immunization card and mothers recall, 9.5% (57) were partially vaccinated and the remaining 1.0% received no vaccination. In all, 63 (10.5%) of children had either received none or only some of the vaccines (<7 vaccines or 15 doses) by the time of the survey (not fully immunized). Nearly all (99.0%) children took Pentavalent 1, Pentavalent 2, OPV 1, OPV 2, Rota 1 and Rota 2. The lowest coverage (92%) was recorded among measles and yellow fever vaccines (Table 2).

**Table 2:** Immunization coverage by immunization card and mothers/caregivers recall, Immunization Coverage Survey, Techiman Municipality, Ghana, 2016

Vaccines	Coverage by card only		Coverage by recall only		Coverage by either card or recall	
	Frequency	%	Frequency	%	Frequency	%
BCG	570	95.0	15	2.5	585	97.5
OPV0	540	90.0	42	7.0	582	97.0
OPV1	579	96.5	15	2.5	594	99.0
OPV2	582	97.0	12	2.0	594	99.0
OPV3	555	92.5	9	1.5	564	94.0
Pentavalent1	579	96.5	15	2.5	594	99.0
Pentavalent2	582	97.0	12	2.0	594	99.0
Pentavalent3	555	92.5	9	1.5	564	94.0
PCV1	579	96.5	12	2.0	591	98.5
PCV2	582	97.0	9	1.5	591	98.5
PCV3	553	92.2	9	1.5	562	93.7
Rota1	579	96.5	15	2.5	594	99.0
Rota2	582	97.0	12	2.0	594	99.0
Measles	537	89.5	15	2.5	552	92.0
Yellow fever	537	89.5	15	2.5	552	92.0
Fully immunized					537	89.5
Partially immunized					57	9.5
Not immunized					6	1.0

### Reasons for immunization failure, according to the mothers’ declarations

Children who were not fully immunized (*n* = 63)], their mothers were asked for possible reasons or contributing factors. Out of the 63 children, 57.1% explained that time of immunization was inconvenient and 14.3% said the place of immunization was unknown and mother being too busy with other tasks (Table 3).

**Table 3:** Reasons for immunization failure as reported by mothers/caregivers, Immunization Coverage Survey, Techiman Municipality, Ghana, 2016

Reason (*N* = 63)	Frequency	%
Place of immunization unknown	9	14.3
Fear of side reaction	3	4.8
Time of immunization inconvenient	36	57.1
Child being ill	4	6.3
Mother too busy	9	14.3
Vaccine not available	2	3.2
Total	63	100.0

### Determinants of immunization status

Table 4 shows child immunization status and its potential predicators among children 12–23 months of age. Using bivariate analysis, immunization status was tested with each of the potential predictors as independent variables. Then, multivariate analysis was performed controlling for all the potential predictors. In all, six factors were found as predictors of child immunization status in the multivariate analysis- age of mother/caregiver, marital status, ethnicity, religion, sex of child and possession of child immunization card. Children of older mothers were more likely to be fully immunized, e.g. the AOR was 0.15 (95%CI 0.05–0.87) when the age was 40–49 years compared to less than 20 years. Children of married couples (AOR = 0.29, 95%CI = 0.13–0.68) were more likely to be fully immunized compared to children of never married/singles mothers. In comparing other ethnic groups to Akan (the main ethnic group), Frafra (AOR = 4.71, 95%CI = 146–15.18) have a higher likelihood of not being fully immunized where as Kusaasi (AOR = 0.09, 95%CI = 0.02–0.51) have a higher likelihood of being fully immunized. Children from the Christian religion (AOR = 0.17, 95%CI = 0.06–0.50) were more likely to be fully immunized compared to children from the Islam religion. Also, in comparing female children to male children, it was found that, female children (AOR = 0.39, 95%CI = 0.19–0.80) were more likely to be fully immunized than their counterpart male children and lastly, children whose immunization cards were not available at the time of study were more likely not to be fully immunized than those children whose immunization cards were available. The AOR was 84.43 (95%CI = 17.04–418.33) when those not having cards was compared to those having cards.

**Table 4:** Odd ratio (bivariate and multivariate logistic regression) of being not fully vaccinated, Immunization Coverage Survey, Techiman Municipality, Ghana, 2016

Variable	Bivariate		Multivariate	
	Crude OR (95% CI)	*P*-value	Adjusted OR (95% CI)	*P*-value
Age of respondent (in years)				
≤19	1.0		1.0	
20–29	0.73 (0.32–1.65)	0.445	0.40 (0.13–1.19)	0.098
30–39	0.66 (0.29–1.50)	0.319	0.69 (0.19–2.54)	0.580
40–49	0.25 (0.07–0.99)	0.048	0.15 (0.05–0.87)	0.035
> = 50	“-”^a^		“-”^a^	
Child’s relation to respondent				
Mother	1.0		1.0	
Not mother	0.94(0.39–2.29)	0.894	0.69 (0.23–2.11)	0.518
Number of children (parity)				
1–2 children	1.0		1.0	
> 2 children	1.31(0.77–2.24)	0.319	1.44 (0.59–3.51)	0.419
Education				
No formal education	1.0		1.0	
Primary school	0.67 (0.30–1.51)	0.337	1.15 (0.37–3.52)	0.813
JSS/Middle school	1.07 (0.56–2.04)	0.845	1.21 (0.46–3.20)	0.698
Senior secondary school	“−”^a^		“−”^a^	
Tertiary education	“−”^a^		“−”^a^	
Marital status				
Never married/single	1.0		1.0	
Married	0.66 (0.39–1.12)	0.127	0.29(0.13–0.68)	0.004
Ethnicity				
Akan (main ethnic group)	1.0		1.0	
Dagaarti	1.26 (0.61–2.59)	0.531	0.53 (0.17–1.68)	0.282
Frafra	2.52 (1.19–5.33)	0.016	4.71 (1.46–15.18)	0.010
Kusaasi	0.45 (0.13–1.53)	0.201	0.09 (0.02–0.51)	0.006
Ewe	“−”^a^		“−”^a^	
Dagomba	2.83 (1.22–6.59)	0.016	1.75 (0.39–7.78)	0.464
Other	“−”^a^		“−”^a^	
Religion				
Islam	1.0		1.0	
Christianity	0.48 (0.27–0.84)	0.010	0.17 (0.06–0.50)	0.001
Traditional	“−”^a^		“−”^a^	
No religion	1.08 (0.40–2.86)	0.885	0.32 (0.06–1.69)	0.180
Occupation				
Farmer	1.0		1.0	
Salary worker	“−”^a^		“−”^a^	
Petty trading	1.03 (0.53–1.98)	0.938	0.60 (0.24–1.54)	0.290
Artisan	1.08 (0.48–2.41)	0.859	0.67 (0.23–2.01)	0.480
Others	1.11 (0.41–3.06)	0.843	1.09 (0.32–3.65)	0.890
Age of child	1.0		1.0	
Age in months (12–23)	1.05 (0.97–1.15)	0.220	1.08 (0.96–1.20)	0.177
Sex of child				
Male	1.0		1.0	
Female	0.60 (0.35–1.02)	0.058	0.39 (0.19–0.80)	0.010
Possession of immunization card				
Yes	1.0		1.0	
No	20.82 (7.50–57.82)	<0.001	84.43 (17.04–418.33)	<0.001

“−”^a^: 100% fully immunized

We found no association between immunization status and the remaining factors (child’s relationship, parity, education, occupation, and age of child). In the multivariate analysis, mothers/caregivers aged 40–49 years, married mothers, Kusaasi ethnic groups, Christian and female child were more likely to be fully immunized. While Frafra ethnic group and no child immunization card were more likely not to be fully immunized.

## Discussion

This study evaluated immunization coverage and its associated factors among children aged 12–23 months in Ghana. Childhood full immunization status was confirmed using child health card and mothers/caregivers recall. Immunization coverage for all 15 vaccines doses ranged 92.0% (Yellow and Measles) to 99.0% (OPV1, OPV2, Penta 1, Penta 2 and Rota 1). Overall, this study found high immunization coverage (89.5%) in the study area. This is about 12.5% higher than the national coverage in Ghana [6].

In Ghana, BCG and OPV0 vaccines are given immediately after birth. If a child missed to take either vaccines immediately, OPV0 can still be taken within first 2 weeks after birth while BCG remains available for taking by child until 1 year of age [5]. Both BCG and OPV0 vaccines had similar coverages of 97.5 and 97.0% respectively. Similarly, OPV1-OPV3, Pentavalent 1 to Pentavalent 3 and PCV1-PCV3 are all given according to the same schedule. Thus, the similarity in coverage among OPV and Pentavalent. However, there was a slight drop in coverage for PCV. This could not be explained but to assume due to vaccine shortage since it is a new vaccine in Ghana. Probably, it is having initial implementation challenges as often with new programs or interventions. The coverages for measles and yellow fever were also similar. Generally, these two vaccines were lower than the coverages of all the other vaccines. The explanation is likely due to the relatively longer time interval between the other vaccines (from birth to 14 weeks) compared to measles and yellow fever vaccines which are given at 9 months after birth. A significant number of mothers/caregivers may forget to bring their children for measles and yellow vaccines. This is consistent with other studies that measles and yellow fever coverages are usually lower compared to other vaccines [10].

Globally, United Nations Children Fund (UNICEF) and Global Alliance for Vaccines and Immunization (GAVI) proposed 80% coverage in Pentavalent3 as a proxy for high immunization coverage [10]. Thus, this study confirms high immunization coverages in Ghana. Immunization coverages are from 84% (OPV3 and PCV3) to 97% (BCG, Pentavalent1 and OPV1) [6]. Similarly, high immunization overages are reported for these particular vaccines in Cameroon and Malawi [10, 11]. We think the high immunization coverages in the current study may be attributed to increased health services delivery such as creation of more outreach immunization sites through the Community-based Health Planning and Services (CHPS) and static centers in the Municipality. In addition, the private health care providers are now involved in the provision of BCG and OPV0 immunization. Thus, increased access to immunization services in general. Moreover, sensitization of health facility managers through the decentralization concept to support immunization including transportation and vaccine storage may have contributed to high immunization coverages in the study area. Despite the coverage improvement, 10.5% of the children were not fully immunized. This is a proxy indicator of immunization defaulting among the children. Mother/caregivers reported time of immunization being inconvenient, place of immunization as unknown, being too busy with other tasks, child being ill, fear of side reaction/effect from vaccination and vaccines not available at health facilities as some of the reasons for not fully immunized. Findings from other studies have reported similar reasons for immunization status [12].

In all, six (6) factors were found to be associated with child immunization status, namely age of mother/caregiver, marital status, ethnicity, religion, sex of child and possession of child immunization card. Using multivariate analysis, age of mother/caregiver, marital status, ethnicity, religion, sex of child and possession of child immunization card were found as predictors of child immunization status. The children with respondent age 40–49 years, married mothers, Kusaasi ethnic groups, Christian and female child were more likely to be fully immunized, while Frafra ethnic group and not having child immunization card were more likely not to be fully immunized.

Junior/Middle school education of respondent compared to no formal education was more likely not to be fully immunized. However, this was not statistically significant. From senior secondary to tertiary education, all children were fully immunized. Increased education is usually expected to improve health seeking behavior positively. Our finding is similar to previous findings of secondary or higher education being a predictor of fully immunization [8, 13–15]. In Ghana, it was found that basic vaccination coverage increased with increasing education of mothers in the 2014 GDHS [6]. We found increased in maternal age corresponded with improved child immunization status. This pattern was observed in other studies conducted in Cameroon and Nigeria. The authors explained that older mothers/caregivers have better knowledge on the effect and relevance of immunization on child health due to previous exposures compared to younger women [11, 16]. In Bangladesh, a similar finding showed that maternal age is essential for child immunization status [17].

Marital status was also found to be a predictor of immunization status. Children with married mothers/caregivers were more likely to be fully immunized. A potential explanation is married respondents are more likely to be psychologically stable compared to single parenting due to trauma, stigma, socioeconomic challenges and unplanned pregnancies. The occupation of mothers/caregivers was not statistically significant. However, all salary workers had their children fully immunized. Previous studies reported that maternal occupation and wealth are predictors of completeness (fully immunized) childhood immunization [18–20]. Salary work provides women with more influence and decision making power compared to non-salary workers or income constrained mothers. This could be the reason for improved child immunization status. In Ghana, the maternity leave policy (leave from work to take care of a newborn) which is enjoyed by salary workers in the formal sector. This “free-time” may be used for child health care including immunization of newborns.

In the multivariate analysis, we found possession of child immunization card to be a predictor of immunization status. Children without immunization cards were more likely not to be fully immunized. In Cameroon, a similar finding was reported that having a child immunization card increased chances of receiving immunization compared to their colleagues [11]. Though the reason for this disparity is unexplained. We think, it may be due to the perceived ill-treatment that mothers will receive from health care providers when they are informed of the lack of child immunization card (due to misplaced, lost or spoiled). Thus, they avoid seeking immunization services for their children.

We also found religion to be a predictor of immunization status. Mothers/caregivers having Christianity as their religion were more likely to be fully immunized. Majority of Muslim are often not formally educated and also not in salary work activities. This may be a potential explanation for the statistical significance found among Christian respondents. In Nigeria, misconception among Muslims has been one of the justifications for no or low immunization uptake. The authors explained immunization activities are perceived to be deliberately designed by outsiders (enemies of Islam) targeted to reduce Muslim population through fortification of vaccines [16]. Misconceptions like this could flow-over to neighboring countries including Ghana.

Ethnicity remains a major factor in participation and acceptance of health services including immunizations. Frafra ethnic group was more likely not to be fully immunized. Mothers/caregivers among the minority in commercial towns are likely to have no formal education, non-salary work, unmarried and other religious denomination rather than Christianity. These challenges and interactions may be the explanation for their children’ less likelihood in completing their immunization.

### Limitation of the study

The mothers recall was a limitation. This is prone to systematic error (recall bias) caused by differences in accuracy of immunization information over a period up to 2 years. Since immunization status and predicting factors were assessed simultaneously, it is impossible to establish cause-effect relationship. Despite this limitation, the results are useful for the Municipality, research community and Ghana.

## Conclusion

In conclusion, immunization status (89.5%) and coverages ranged 92 to 99% of the 15 vaccine doses. This is high compared to national as well as GAVI and UNICEF target of 80%. In all, six factors were found as predictors of child immunization status - age of mother/caregiver, marital status, ethnicity, religion, sex of child and possession of child immunization card. About 10.5% of the children were not fully immunized. The reasons reported for children not fully immunized in the study area were: time of immunization being inconvenient; place of immunization unknown; mother too busy with other tasks; child being ill; fear of side reaction; and inadequate vaccines. Problems of not fully immunized persists and needs urgent attention. Education on immunization should be intensified by health providers. In addition, reliable registers for immunization services will improve data quality and accuracy as well as defaulters’ identification and tracing. Moreover, disadvantaged populations should continue to be reached with immunization services using out-reach activities.

## Abbreviations

BCG: Bacillus Calmette-Guerin
CHPS: Community-based health planning and services
EPI: Expanded programme on immunization
GAVI: Global alliance for vaccines and immunization
GDHS: Ghana demographic and health survey
GHS: Ghana health service
JHS: Junior high school
OPV: Oral polio vaccine
PCV: Pneumococcal vaccine
SHS: Senior high school
UNICEF: United Nations children’s fund
WHO: World health organization
